# Negative Life Events and Antenatal Depression among Pregnant Women in Rural China: The Role of Negative Automatic Thoughts

**DOI:** 10.1371/journal.pone.0167597

**Published:** 2016-12-15

**Authors:** Yang Wang, Xiaohua Wang, Fangnan Liu, Xiaoning Jiang, Yun Xiao, Xuehan Dong, Xianglei Kong, Xuemei Yang, Donghua Tian, Zhiyong Qu

**Affiliations:** 1 School of Social Work, University of Illinois at Urbana-Champaign, Champaign, Illinois, United States of America; 2 School of Social Development and Public Policy, China Institute of Health, Beijing Normal University, Beijing, China; 3 School of Social Development and Public Policy, Beijing Normal University, Beijing, China; 4 Maternal and Child Health Hospital in Mianzhu County, Deyang, Sichuan, China; 5 Teacher Education College, Sichuan Normal University, Chengdu, Sichuan, China; University of Rochester, UNITED STATES

## Abstract

**Background:**

Few studies have looked at the relationship between psychological and the mental health status of pregnant women in rural China. The current study aims to explore the potential mediating effect of negative automatic thoughts between negative life events and antenatal depression.

**Methods:**

Data were collected in June 2012 and October 2012. 495 rural pregnant women were interviewed. Depressive symptoms were measured by the Edinburgh postnatal depression scale, stresses of pregnancy were measured by the pregnancy pressure scale, negative automatic thoughts were measured by the automatic thoughts questionnaire, and negative life events were measured by the life events scale for pregnant women. We used logistic regression and path analysis to test the mediating effect.

**Results:**

The prevalence of antenatal depression was 13.7%. In the logistic regression, the only socio-demographic and health behavior factor significantly related to antenatal depression was sleep quality. Negative life events were not associated with depression in the fully adjusted model. Path analysis showed that the eventual direct and general effects of negative automatic thoughts were 0.39 and 0.51, which were larger than the effects of negative life events.

**Conclusions:**

This study suggested that there was a potentially significant mediating effect of negative automatic thoughts. Pregnant women who had lower scores of negative automatic thoughts were more likely to suffer less from negative life events which might lead to antenatal depression.

## Background

The prevalence of antenatal depression varies from 7.4% to 50.0% worldwide, and from 5.5% to 23.1% in China [1–7]. The prevalence of depression among pregnant women is higher than that among postpartum women, and it is higher among pregnant women in middle and late pregnancy [5, 8]. Antenatal depression is a debilitating experience which can lead to many problems and sequelae. For example, depressed pregnant women may experience multiple conflicting roles, insufficient social support, uncertainty about future life, instability of emotion, and the discontent of body image. Moreover, there can be a risk of preterm birth and obstetric complications. And the newborns’ and husbands’ mental and physical health can also be threatened by their mothers’ and wives’ depression as well [9–16].

The predictors of antenatal depression include several socio-demographic and health behavior factors such as young or old age, low educational background, and low socio-economic status. Besides, antenatal depression is always related with threatening life events, such as housing problems, financial difficulties, and marital problems [17]. And negative life events contribute more to antenatal depressive symptoms than other socio-demographic factors do [18, 19]. Moreover, there are psychosocial factors such as stress, low social support, and low optimism level that can lead to an increased antenatal depressive level [7, 20–22].

Apart from those listed above, automatic thoughts are also believed to have a reciprocal relationship with depression, indicating that automatic thoughts can be the outcome of depression, and it can also have an impact on depressive level [23]. Automatic thoughts reflect one’s underlying core beliefs. If some events, which challenge one’s core beliefs, take place, the stream of negative automatic thoughts may run through one’s mind, and it will cast a negative interpretation of the events. The negative interpretation may increase the stress level and cause depressive symptoms [24–29]. That is consistent with the vulnerability model, which postulates that negative automatic thoughts play a mediating role between negative life events and depression [23]. This mediating effect has been examined in the study among adolescents [30]. It has also been applied in practice. Some interventions, which employed cognitive-behavioral therapy principles, used this mediating effect with their design of targeting and altering negative automatic thoughts, and building more adaptive automatic thoughts among pregnant women [24, 31].

Rural pregnant women used to be believed to have a lower depressive level in some studies, because they are supposed to have stronger family support and community connections, which can protect them from the risk of perinatal depression[32, 33]. Conversely, some studies showed conflicting findings. According to a quantitative study conducted in four provinces of China from 2001 to 2005, which included 63004 adult participants from both rural and urban sampling sites, there was a higher prevalence of depressive disorders among rural residents than that among urban residents [34, 35]. Further, some studies illustrated the difficulties encountered by rural pregnant woman. Pregnant women in rural areas could suffer from the isolation from their spouses, and the mental health services in rural areas were limited [36–38].

In present rural China, this issue is more complex. There has been a trend that many male laborers prefer to go to urban areas as migrant workers since the 1980s. Since the dual division system of city and countryside did not allow migrant workers to relocate the whole family with them to urban areas, many wives of the migrant workers had to be left behind in their rural residences, doing farming work and taking care of families. That created a burden on the daily life of the left-behind wives, and led to their worse mental health status [39, 40].

This is the first study that aimed to assess the mediating effect of negative automatic thoughts between negative life events and antenatal depressive symptoms among rural pregnant women in China. We postulate that among rural pregnant women in China, negative life events and antenatal depression are mediated by negative automatic thoughts.

## Materials and Methods

### Study design and participants

The study is a cross-sectional hospital-based survey, and it was conducted in Mianzhu County in Sichuan Province and in Gaobeidian County in Hebei Province, China. Ethical approval and consent processes were obtained from the institutional review board of the School of Social Development and Public Policy at Beijing Normal University.

Ten volunteers, who were graduate students majoring in psychology, received a two-day training to be qualified interviewers, and they investigated the participants. In Mianzhu County, the data were collected twice, once in June 2012 and once in October 2012. The data collection in October interviewed a new population of pregnant women. In Gaobeidian County, the data were collected only in October. Each collection lasted for approximate eight days. A self-rating questionnaire was distributed to pregnant women who were receiving routine prenatal care at the hospitals. The women completed the self-reporting questionnaires in approximate 30 minutes during their waiting for a routine antenatal check-up. A signature on the consent form was regarded as a sensitive issue in local cultural context, and participants could reject the investigation because of it. Moreover, this anonymous study was little risk to participants. Thus, an informed oral consent was obtained from each study participant instead of a written consent. The study had been explained to each participant by volunteers, and the volunteers had answered the participants’ questions prior to asking for their permission to conduct the investigation. Participation was entirely voluntary, and each participant had the right to withdraw or to refuse to provide information at any time during the study.

The participants from Mianzhu County got interviewed at Mianzhu People's Hospital and Mianzhu Maternal and Child Health Hospital, where around 70% of the pregnant women in this county received antenatal care and post-delivery services. The participants from Gaobeidian County got interviewed at Gaobeidian County’s Hospital, where nearly 50% of the pregnant women in this county received antenatal care and post-delivery services. The two hospitals where this study was conducted are the top ranked hospitals in the two counties.

### Measurements

Antenatal depression was rated by the Edinburgh postnatal depression scale (EPDS).EPDS is a 10-item self-reporting instrument, used to screen for antenatal depressive symptoms, and it is most frequently used in epidemiological research about antenatal depression [41, 42]. The items are rated on a 0 to 3 scale, yielding a total score range of 0 to 30. The items focus on the cognitive and affective features of depression. The EPDS has been employed in studies of China, and it was reliable in measuring prenatal and postnatal women's depression [43–46]. The Cronbach’s alpha coefficient of the Chinese version of the EPDS in this study was 0.689. A cut off point score of ≥13 was used to determine depressive symptoms in the bivariate correlation analysis, as was recommended in a previous study [47, 48].

The perceived pressures of pregnancy were rated by an 11-item self-assessment scale which is a short-form of the pregnancy pressure scale (PPS) developed by Zhanghui Chen et al. The Cronbach's alpha coefficient was reported to be 0.84 [49]. This scale contains three subscales: pressure from identification of the parent’s role, pressure from the concerns of maternal and child health, and pressure from the change of body shape or physical activities. The scale measures the perceived stress of major pregnancy-related events by using a 4-point Likert scale from 1 (none or little) to 4 (high). The total score is used as an index of perceived stress. Possible stressful experiences include: fears about important people disliking the baby, concerns about reduced leisure time with a baby, fears about the safe delivery of the baby, anxiety about birth defects, fears about complications during delivery, fears about pain during delivery, concerns about changes in body shape, fears about competence as a mother, fears about the negative impact of the baby on the marital relationship, concerns about providing a healthy living environment to the baby, and other pregnancy-related stresses.

The negative automatic thoughts were measured by the automatic thoughts questionnaire (ATQ) [43, 50]. This 30-item questionnaire is devised to measure the frequency of the occurrence of automatic negative thoughts associated with depression. Four aspects of automatic thoughts are measured. They are personal maladjustment and the desire for change, negative self-concepts and negative expectations, low self-esteem, and helplessness. The responses of every question are provided on a 5-point scale from not at all (1) to all of the time (5). A high total score indicates more frequent negative thoughts. The questionnaire has been used in many studies about depression in China and elsewhere. The studies in China found that a change of the scores of the ATQ was significantly consistent with the change of the scores of the Self-Rating Depression Scale [51–53]. In the current study, the examination of the internal consistency of the ATQ yielded a coefficient alpha of 0.93, which suggested a high level of internal consistency. The ATQ-30 appears to be a reliable measure of automatic thoughts in depression.

The negative life events were measured by the life events scale for pregnant women (LESPW) [54]. This scale is self-rating, and it is designed specifically for pregnant women. It includes life events which lead to different levels of sensation, and the events concern family life, work and study, social relationships, etc. There are 53 events, and they are divided into two groups which involve subjective events (SE) and objective events (OE) separately. The OE are also divided into three levels by the extent they impact the emotion of the pregnant women, and they are grouped into OE1, OE2, OE3. The current study used the OE, because the SE are more related with subjective feelings, while we have used other special tools to measure them, which are negative automatic thoughts, stress, and antenatal depression. Thus, we only need the events of in the OE as the events that can lead to stress and depression. Besides, the current study only employed OE2 and OE3, because the events included in OE2 and OE3 have greater significance on sensation to people, and they can have a greater and long- lasting impact on pregnant women’s depressive level. Thus, in order to decrease the influence brought by the temporality of events, we only chose events in OE2 and OE3. The events are all negative life events, which fit well with the objective of this study. A total of 34 life events in OE2 and OE3 were used. In the current study, the scale showed a good internal consistency (Cronbach's Alpha = 0.60).

The socio-demographic and health behavior factors include the participants’ age (18–24 years; 25–29 years; ≥30 years), region (Mianzhu; Gaobeidian); level of education (middle school or lower; high school; college or above), parity (primigravida; others), monthly family income (USD <326; USD 326–816; USD ≥816), living site (village; city), planned pregnancy (yes; no), body mass index (BMI) (overweight is defined as BMI≥23 among Asian population by WTO), the stage of gestation (0–28 weeks; >28 weeks), employment (unemployed; part-time job; full-time job), sleep quality (good; fair; poor), smoking history (yes; no), alcohol use history (yes; no), whether the husband is a migrant worker (yes; no).

### Statistical analysis

SPSS 17.0 (SPSS Inc, Chicago, IL) was used for statistical analysis. A descriptive analysis was performed for the socio-demographic and health behavior characteristics (region, age, level of education, monthly household income, living site, employment, planned pregnancy, BMI, gestation stage, parity, sleep quality, smoking history, alcohol use history, whether the husband is a migrant worker). Chi-square tests and a bivariate correlate analysis were performed to examine the correlation between the outcome variable and the independent variables, and the socio-demographic and health behavior factors. Logistic regression was used to test the socio-demographic and health behavior factors, negative life events, stresses of pregnancy, and negative automatic thoughts as predictors of antenatal depression. All of the estimates were accompanied by odds ratios (OR) and 95% confidence intervals.

AMOS 17.0 was used for the path analysis. Negative life events, negative automatic thoughts, stresses of pregnancy, and antenatal depression were included in the models. The comparative fit index (CFI), the incremental fit index (IFI), and the root mean square error of approximation (RMSEA) with 90% confidence intervals were used to estimate the model fit.

## Results and Discussion

### Results

The mean age of the participants was 25.49 (min = 18.0, max = 42.0; standard deviation [SD] = 3.85) and nearly half (47.3%) of the participants were 18–24 years old. Nearly half of the pregnant women (47.8%) completed middle school or less. Most of the pregnant women (82.0%) had a family monthly income lower than 816 USD. Most of the pregnant women (86.9%) were unemployed during their pregnancy. Approximately half (50.5%) of the pregnancies were planned. More than half (58.7%) of the women were primigravida, and most of them (70.8%) had been pregnant for more than 28 weeks. The BMI of most of them (74.5%) were over 23. More than half (52.7%) of the pregnant women had a fair quality of sleep, and there were smaller percentages of pregnant women with a good quality of sleep (39.2%) or a poor quality of sleep (8.1%). Almost all (98.2%) of the pregnant women had no smoking history, and almost all (94.3%) of the pregnant women had no history of alcohol use. Over half (54.5%) of the pregnant women’s husbands were migrant workers (Table 1).

**Table 1:** Description of socio-demographic and health behavior characteristics, scores of AND, stresses of pregnancy, NLE, and NAT of the participants (n = 495).

Socio-demographic and Health Behavior characteristics	N	%
Region		
Mianzhu	249	50.3
Gaobeidian	246	49.7
Age		
18–24	236	47.3
25–29	175	35.1
≥30	88	17.6
Education		
Middle school or lower	239	47.8
High school	165	33.0
College or above	96	19.2
Monthly family income(USD)		
<326	164	33.1
326–816	242	48.9
≥816	89	18.0
Living site		
Village	396	80.0
City	98	20.0
Employment		
Unemployed	424	86.9
Part-time job	18	3.7
Full-time job	46	9.4
Planned pregnancy		
Planned	244	50.5
Unplanned	239	49.5
BMI		
<23	124	25.5
≥23	363	74.5
Parity		
Primigravida	293	58.7
Others	206	41.3
Gestation stage		
≤28weeks	130	29.2
>28weeks	323	70.8
Sleep quality		
Poor	40	8.1
Fair	261	52.7
Good	194	39.2
Smoking history		
Non	486	98.2
Yes	9	1.8
Alcohol use history		
Non	466	94.3
Yes	28	5.7
Husband is a migrant worker		
Yes	261	54.5
Non	218	45.5
Depression		
<13	427	86.3
≥13	68	13.7

Note: some variables have missing values.

The mean score of negative automatic thoughts was 39.07 (min = 30, max = 99; SD = 10.71), and the median score was 35.00. The mean score of negative life events was 105.31 (min = 0, max = 515; SD = 100.27), and the median score was 84.00. The mean score of stresses of pregnancy was17.11 (min = 10, max = 40; SD = 4.70).

The mean score of antenatal depression was 8.71 (min = 0, max = 25; SD = 3.91), and the median score was 9.00. The EPDS cutoff point recommended by Rubertsson’s study is a score of ≥13. According to this criterion, the prevalence of major depressive symptoms in the current study was 13.7% [48].

Table 2 displays the bivariate correlations between antenatal depression and the socio-demographic and health behavior factors in the study. Antenatal depression was only significantly correlated with the sleep quality (p = 0.001), while other variables didn’t have a significant relationship with antenatal depression.

**Table 2:** The prevalence of antenatal depression by socio-demographic and health behavior factors.

	Depression	Depression	P
	Score<13 n(%)	Score≥13 n(%)	
Region			
Mianzhu	219(88.0)	30(12.0)	0.272
Gaobeidian	208(84.6)	38(15.4)	
Age			
18–24	196(83.8)	38(16.2)	0.097
25–29	158(90.8)	16(9.2)	
≥30	73(83.9)	14(16.1)	
Education			
Middle school or lower	203(86.4)	32(13.6)	0.692
High school	139(84.8)	25(15.2)	
College or above	85(88.5)	11(11.5)	
Monthly family income(USD)			
<326	136(84.0)	26(16.0)	0.516
326–816	208(87.0)	31(13.0)	
≥816	79(88.8)	10(11.2)	
Living site			
Village	341(86.1)	55(13.9)	0.873
City	85(86.7)	13(13.3)	
Employment			
Unemployed	364(86.7)	56(13.3)	0.449
Part-time job	14(77.8)	4(22.2)	
Full-time job	38(82.6)	8(17.4)	
Planned pregnancy			
Planned	213(88.4)	28(11.6)	0.162
Unplanned	199(84.0)	38(16.0)	
BMI			
<23	102(83.6)	20(16.4)	0.401
≥23	312(86.7)	48(13.3)	
Gestation stage			
≤28weeks	111(85.4)	19(14.6)	0.971
>28weeks	278(86.1)	45(13.9)	
Parity			
Primigravida	255(87.6)	36(12.4)	0.282
Others	171(84.2)	32(15.8)	
Sleep quality			
Poor	28(70.0)	12(30.0)	0.001
Fair	222(85.1)	39(14.9)	
Good	177(91.2)	17(8.8)	
Smoking history			
Non	418(86.0)	68(14.0)	0.227
Yes	9(100.0)	0(0.0)	
Alcohol use history			
Non	405(86.9)	61(13.1)	0.076
Yes	21(75.0)	7(25.0)	
Husband is a migrant worker			
Yes	230(88.1)	31(11.9)	0.145
Non	182(83.5)	36(16.5)	

Table 3 displays the results of the bivariate correlations. Antenatal depression was significantly and positively correlated with negative life events (r = 0.33, p<0.01), stresses of pregnancy (r = 0.44, p<0.01), and negative automatic thoughts (r = 0.54, p<0.01). And the results show each of the four variables was significantly correlated with the other three.

**Table 3:** Bivariate correlate analysis of the scores of antenatal depression, stresses of pregnancy, negative life events and negative automatic thoughts.

	Antenatal depression	Stresses of pregnancy	Life events scale for pregnant women	Negative automatic thoughts
Antenatal depression(n = 495)	1	0.44**	0.33**	0.54**
Stresses of pregnancy(n = 481)		1	0.27**	0.49**
Life events scale for pregnant women(n = 491)			1	0.37**
Negative automatic thoughts (n = 495)				1

**P<0.01

Table 4 shows the results of logistic regression for predicting the variables of antenatal depression. According to the results, significant predictive variables included sleep quality, the stresses of pregnancy, and negative automatic thoughts. Among them, higher scores of negative automatic thoughts (OR, 1.08; 95% CI, 1.04–1.11) and a higher level of stress of pregnancy (OR, 1.12; 95% CI, 1.04–1.21) were significantly related with a higher level of antenatal depression.

**Table 4:** Logistic regression analysis of socio-demographic and health behavior factors and life events of pregnancy, stresses of pregnancy, and negative automatic thoughts for antenatal depression.

	Independent variable	OR(95% CI)
Region	Mianzhu: Gaobeidian	0.51(0.24–1.09)
Age	Ref: ≥30	
	18–24	1.22(0.39–3.76)
	25–29	0.51(0.16–1.61)
Education	Ref: College or above	
	Middle school or lower	0.42(0.13–1.32)
	High school	0.59(0.19–1.82)
Monthly family income(USD)	Ref: ≥816 USD	
	<326	1.51(0.53–4.32)
	326–816	1.00(0.39–2.59)
Living site	Village: City	1.17(0.42–3.28)
Employment	Ref: full-time job	
	Unemployed	0.90(0.29–2.76)
	Part-time job	1.79(0.29–11.20)
Planned pregnancy	Planned: unplanned	0.89(0.44–1.78)
BMI	<23:≥23	1.42(0.62–3.26)
Parity	Primigravida: Others	0.49(0.21–1.14)
Gestation stage	0-28weeks:>28weeks	1.65(0.74–3.68)
Sleep quality	Ref: Poor	
	Good	0.25(0.08–0.84)
	Fair	0.43(0.14–1.30)
Smoking history	Non: Yes	5.273E8(0.00–0.00)
Alcohol use history	Non: Yes	0.82(0.21–3.26)
Husband is a migrant worker	Non: Yes	1.38(0.66–2.85)
Life events of pregnancy		1.00(1.00–1.01)
Stresses of pregnancy		1.12(1.04–1.22)
Negative automatic thoughts		1.08(1.04–1.11)

**Fig 1:**
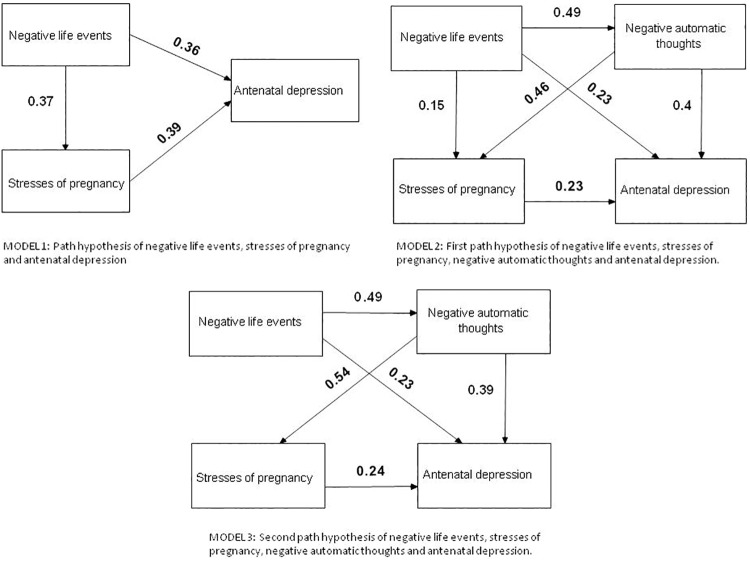
Path models examining associations of negative life events, stresses of pregnancy, negative automatic thoughts and antenatal depression.

Based on the regressive model above, we tested three path analytic models that predicted antenatal depression (Fig 1). In Model 1, negative life events and stresses of pregnancy were used as the predictors, and antenatal depression was the parameter estimate. In Model 2 and 3, negative automatic thoughts were included as a predictor of antenatal depression, and this was also a parameter estimate of negative life events in Model 2 and 3.

In Model 1, there are three hypotheses. The first one was that negative life events had a direct effect on antenatal depression. The second one was that stress had a direct effect on antenatal depression. The third one was that stress played a mediating role between negative life events and antenatal depression. The fit indexes were good for this model (χ^2^ (206, n = 275) = 608.265, p<0.001; χ^2^/df = 2.95; RMSEA = 0.063, IFI = 0.827, CFI = 0.824).

In Model 2, there are four hypotheses. The first hypothesis was that negative life events had a direct effect on antenatal depression as important predictors. The second hypothesis was that negative automatic thoughts and stresses of pregnancy had direct effects on antenatal depression separately. The third hypothesis was that stress had a mediating effect between negative life events and antenatal depression, and it had a mediating effect between negative automatic thoughts and antenatal depression. The fourth hypothesis was that negative automatic thoughts had a mediating effect between negative life events and antenatal depression. So we included negative automatic thoughts into Model 2. This Model revealed significant direct and indirect paths from negative life events to antenatal depression, and the fit of the model was better than it of Model 1 (χ^2^ (344, n = 434) = 955.859,p<0.001; χ^2^/df = 2.78; RMSEA = 0.060, IFI = 0.859, CFI = 0.857).

We moved away the path between negative life events and stress, and created Model 3. The significant paths of this model are illustrated in Fig 1. The model fit was good (χ^2^ (345, n = 434) = 960.604, p<0.010; χ^2^/df = 2.78; RMSEA = 0.060, IFI = 0.858, CFI = 0.856).

The path models demonstrate that antenatal depression was influenced by negative life events, stresses of pregnancy and negative automatic thoughts. In Model 1, the model fit indices indicated that this model was good. In Model 2, after adding negative automatic thoughts, the indices were improved. In Model 3, after moving away the path between negative life events and stresses of pregnancy, the indices did not change much, and the final model was simplified, indicating that the final model was better.

We assessed how well the three variables could predict antenatal depression in Model 3. The results showed that negative automatic thoughts had a greater direct effect (0.39) than negative life events (0.23) and stresses of pregnancy (0.24) did. The general effect of negative automatic thoughts (0.51) was greater than the effects of negative life events (0.42) and stresses of pregnancy (0.24).

### Discussion

To our knowledge, this is the first study examining the mediating effect of automatic thoughts between negative life events and antenatal depression in rural China. The results of this study proved the hypothesis that negative automatic thoughts play an important mediating role between negative life events and antenatal depression.

The results indicate that there is more than a direct effect between negative life events and antenatal depression [55]. This result is in agreement with Beck’s cognitive behavioral theory [26]. Previous studies have proved the significant mediating role of automatic thoughts among migrant populations, children and undergraduate students [23, 52, 56]. Moreover, a culturally sensitive cognitive-behavioral therapy was implemented among Chinese pregnant women. It also declared that the key to that intervention was to target the irrational thoughts which were relevant to the life stories told by the pregnant women. And then replace the irrational thoughts with more positive ones [57]. This current study provided further evidence that for pregnant women in rural China, negative automatic thoughts were also significantly and positively associated with their antenatal depression. From the results of the path analysis, this study also found that by comparison with negative life events, negative automatic thoughts had larger direct and general effects on antenatal depression.

Except for sleep quality, the current study did not find any other socio-demographic and health behavior factor which was associated significantly with antenatal depression. The result contradicts some previous studies, which found that the risk factors of antenatal depressive symptoms included lower income, lower education, smoking, alcohol abuse, and single status. Some studies in East Asia found that younger age and unemployment could also contribute to antenatal depression [7, 10, 20–22, 58]. This may be because our sample included a large proportion of pregnant women who were of young ages between 18–29 years old (82.4%). Most of them had educational backgrounds lower than college (80.8%), had monthly family incomes of less than 816 USD (82.0%), and most of them were unemployed (86.9%). The similar characteristics impair the powerfulness of socio-demographic and health behavior factors as predictors.

Previous studies found that the lack of partner support was among the strongest predictors of depression during pregnancy [59, 60]. However, in the current study, there was no significant difference in the depressive level between normal pregnant women who had their husbands around and those whose husbands were migrant workers. It is also known that in this study, over half of the migrant workers (53.2%) visited home at least once a week, and 76.9% of migrant workers contacted their wives every day. It can be drawn that most migrant workers would have a frequent contact with their wives, and this would allow them to provide a good emotional support to their wives. Besides, take financial difficulty, which is another factor associated with perinatal depression, into consideration, since migrant workers could bring the family with more financial income, that could help to decrease their wives’ financial stress and antenatal depressive level [61].

It is still inconvenient for people in rural China to seek for medical treatment for psychological illness, as a result of a severe lack of medical resources [62]. What makes it worse, since the stigma towards people with psychological problems still exists, some depressed pregnant women might hesitate to seek for treatment and assistance. Additionally, in order to avoid the risk of medication, some Asian pregnant women are more hesitant to take antidepressants such as SSRIs [63]. Thus, involving the medical service and socio-cultural background in future studies will help to explore the feature of cognition style of the pregnant women in rural China.

There are several limitations of this study. First, this study mainly focused on the variable of automatic thoughts. However, as the traditional society in rural China is experiencing great changes in many aspects, not much is known about the situation of community support, family support, marital conflicts, and other external environmental factors regarding pregnant women [64, 65]. Thus, without having a deeper consideration about those important issues, our study couldn’t uncover more of the characteristics related with the cognition style of Chinese rural pregnant women. Second, as a cross-sectional study, this current study couldn’t draw a causal relationship between automatic thoughts and antenatal depression among the participants.

## Conclusions

In conclusion, negative automatic thoughts have a potential important mediating effect between negative life events and antenatal depression.

## Supporting Information

S1 File This file provides the minimal data set used for the analysis presented in this article. (SAV)
